# Role of single‐nucleotide polymorphism microarray in the classification of BAP1‐inactivated melanocytic tumours

**DOI:** 10.1111/his.15434

**Published:** 2025-02-20

**Authors:** Joseph S. Durgin, Nicholas A. Zoumberos, Taylor Novice, Douglas R. Fullen, Alexandra C. Hristov, Lori Lowe, Rajiv M. Patel, Paul W. Harms, Aleodor A. Andea, Scott C. Bresler

**Affiliations:** ^1^ Department of Dermatology University of Michigan Ann Arbor Michigan USA; ^2^ Department of Pathology University of Arkansas Little Rock Arkansas USA; ^3^ Department of Dermatology University of Arkansas Little Rock Arkansas USA; ^4^ Department of Pathology University of Michigan Ann Arbor Michigan USA; ^5^ Cutaneous Pathology WCP Laboratories Inc. Maryland Heights Missouri USA; ^6^ Rogel Cancer Center University of Michigan Ann Arbor Michigan USA; ^7^ Roswell Park Comprehensive Cancer Center Department of Pathology Buffalo New York USA

**Keywords:** BAP1‐inactivated melanocytoma, chromosomal microarray analysis, melanoma, single nucleotide polymorphism microarray

## Abstract

**Aims:**

BAP1‐inactivated melanocytic tumours (BIMTs) occur sporadically and in association with a familial tumour predisposition syndrome involving germline mutations in the BRCA1‐associated protein‐1 (*BAP1*) gene. Here we report the clinical features, histopathologic findings, and chromosomal copy number data of 19 BAP1‐inactivated melanocytomas (BIMs) and compare their features to those of five BAP1‐inactivated melanomas.

**Methods:**

We retrospectively searched the Department of Pathology archives and EMERSE (Electronic Medical Record Search Engine) for BIMTs that had undergone single‐nucleotide polymorphism (SNP) microarray testing. Clinical history/follow‐up data, detailed histopathologic features, and SNP microarray results were recorded.

**Results:**

Overall, four patients (4/13) with BIMs and available clinical history had features suspicious for a germline *BAP1* aberration. In BIMs (19 cases), the BAP1‐inactivated component showed variable cytologic features, including epithelioid (predominant), rhabdoid, plasmacytoid, and nevoid morphologies. Sentinel lymph node biopsy was negative in all (6/6) patients in which this procedure was performed. No patient diagnosed with a BIM with available clinical follow‐up (18/19; mean 38 months) experienced an adverse event. While the histologic appearances of the five melanomas varied, one case resembled a BIM. The median mitotic count was 1/mm^2^ (range 0–6 mm^2^) in BIMs compared to 3/mm^2^ (range 1–4/mm^2^) in melanomas (*P* = 0.04). The median number of copy number alterations (CNAs) was two (range 0–6) in indolent cases versus seven (range 6–10) in melanomas (*P* = 0.0005). The most common molecular aberration after loss of 3p21 was heterozygous loss of the *CDKN2A* locus, which unlike homozygous loss has not been associated with melanoma.

**Conclusion:**

While most BIMs appear to have a favourable prognosis, even those with multiple CNAs, they deserve careful integration of all clinical and pathologic findings. Although not fully diagnostic, a mitotic count of ≥3/mm^2^ and ≥6 CNAs in the appropriate context is supportive of a diagnosis of BAP1‐inactivated melanoma.

## Graphical abstract

Here we report the features of 24 BAP1‐inactivated melanocytic tumours including 19 BAP1‐inactivated melanocytomas (BIMs) and five BAP1‐inactivated melanomas. BIMs have a favourable prognosis, even those with multiple copy number aberrations (CNAs). Although not fully diagnostic, a mitotic count of ≥3/mm^2^ and ≥6 CNAs support a diagnosis of BAP1‐inactivated melanoma.

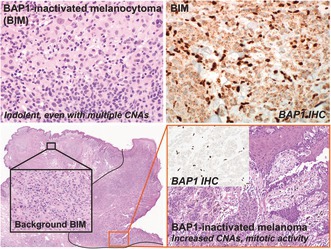

**Abbreviations**
AST: atypical Spitz tumour
BAP1: BRCA1‐associated protein‐1
BIM: BAP1‐inactivated melanocytoma
BIMT: BAP1‐inactivated melanocytic tumour
CNA: copy number alteration
CR: complete response
DPN: deep penetrating nevus
FISH: fluorescence in situ hybridization
HMZ: homozygous
IHC: immunohistochemistry
MIP: molecular inversion probe
NA: not available
NE: not evaluable
NGS: next generation sequencing
NP: not performed
SEM: standard error of the mean
SLNB: sentinel lymph node biopsy
SNP: single nucleotide polymorphism
T‐VEC: talimogene laherparepvec

## Introduction

While most benign melanocytic nevi have single driver mutations and sometimes a low number of copy number alterations (CNAs), melanomas tend to have multiple pathogenic mutations and CNAs.^1^, ^2^ Recently, the classification of melanocytic lesions has evolved to include the category of ‘melanocytoma,’ which refers to tumours that are “genetically intermediate lesions that contain more than one driver mutation, and are usually benign.”^3^ Often, melanocytomas are combined lesions in which a clonal expansion of phenotypically distinct cells, driven by an additional pathogenic mutation, arises within or adjacent to a precursor nevus.^4^ For these uncommon intermediate lesions, the risks of recurrence and progression remain incompletely understood.

BRCA1‐associated protein‐1 (*BAP1*)‐inactivated melanocytomas (BIMs) are a recently defined subset of so‐called intermediate melanocytic neoplasms. These lesions result from two distinct genetic hits: an activating mutation in the MAPK pathway (often in *BRAF*) and an inactivation of the *BAP1* gene at 3p21.^5^ BIMs were originally described in patients with germline inactivating *BAP1* mutations, which cause an autosomal dominant tumour predisposition syndrome with increased risk of uveal melanoma, mesothelioma, meningioma, renal cell carcinoma, and cutaneous melanoma.^6^, ^7^, ^8^ Clinically, BIMs commonly present as pink, tan, or flesh‐coloured papules, plaques or nodules, sometimes with a translucent quality.^9^, ^10^ The majority of BIMs occur sporadically, but the presence of multiple BIMs in the same patient should prompt suspicion for a germline *BAP1* mutation.^11^

Upon histologic evaluation, BIMs classically demonstrate a biphenotypic cytomorphology consisting of both bland ordinary nevomelanocytes and an associated *BAP1*‐inactivated clonal expansion of melanocytes with epithelioid features.^10^ Cases with only an epithelioid component have also been reported.^10^ Prior to the discovery of their distinctive genetics, it is likely that BIMs were commonly classified as senescent nevi due to their nuclear enlargement and pleiomorphism,^12^ or diagnosed as atypical Spitz tumours (ASTs) or even nevoid melanomas due to their lack of maturation and frequent mitotic activity.^13^

BAP1 is a deubiquitinase that functions predominantly in the nucleus, where it regulates transcription, DNA repair, and DNA replication.^14^ BAP1 demonstrates a tumour suppressor function, evident both in the tumour predisposition syndrome and in global and tissue‐specific knockouts in mouse models.^15^, ^16^ In response to mutagenic stimuli, BAP1 coordinates double‐stranded break repair.^17^ Indeed, BAP1‐negative cells show an increased frequency of chromosomal breaks after exposure to ionizing radiation.^17^ Commonly, inactivating mutations in *BAP1*, which are seen in a wide variety of tumour types, produce protein truncations with loss of its nuclear localization signal, which sequesters the protein in the cytoplasm, although complete gene deletions or missense mutations are also observed.^14^

Although melanomas with BAP1 deletion have been reported,^10^, ^11^, ^18^, ^19^ including those arising in BIMs or common acquired nevi,^19^, ^20^ the vast majority of BIMs reported in series to date have had an indolent clinical course.^9^, ^10^, ^21^ In Donati *et al*., of 21 patients with BIMs who had follow‐up, only one patient had an isolated locoregional lymph node metastasis without further progression.^9^ Similarly, Yeh *et al*. had nine patients with BIMs and follow‐up ranging from 4 to 27 months, and all showed no evidence of disease.^10^ That said, the available published data are still sparse, and it remains an important question whether ancillary tests such as single‐nucleotide polymorphism (SNP) / comparative genomic hybridization microarray and fluorescence *in situ* hybridization (FISH) might be useful in these histologically ambiguous tumours.^22^, ^23^, ^24^ Given their distinctive pathogenesis, BIMs require further study to better characterize their expected clinical course, comparison to other categories of melanocytomas, and correlation of outcome with genetic information. Here we describe 19 BIMs and five BAP1‐inactivated melanomas with attention to their histologic features, copy number changes by SNP microarray analysis, and clinical outcomes.

## Materials and Methods

### Case selection

This project was approved by the University of Michigan Institutional Review Board (HUM00045834 and HUM00233671). Informed consent was not required given the retrospective nature of the study and minimal potential risk to the subjects, and the project was performed according to the Declaration of Helsinki. The archives of the Department of Pathology at the University of Michigan and EMERSE (Electronic Medical Record Search Engine) were initially searched retrospectively for all diagnostically challenging or ambiguous melanocytic neoplasms from the period between 2014 and 2024 for which SNP microarray had been performed to aid in the diagnosis.^25^ All lesions with evidence of copy number loss including the *BAP1* locus on chromosome 3p and which showed evidence of BAP1 loss by immunohistochemistry (IHC) were selected for further study. Tumours without loss of 3p but with loss of BAP1 by IHC as well as tumours with loss of 3p but with retained BAP1 expression by IHC which showed typical histopathologic features of a BIM (a biphasic melanocytic neoplasm with a predominant epithelioid phenotype and prominent cell borders with patchy lymphocytic inflammation) were also included. Atypical cellular blue nevi and melanomas ex blue nevus were excluded. Demographic data, comorbidities, family history, and follow‐up were obtained from the electronic medical record of the University of Michigan or the referring institution, if available. An adverse event was defined as recurrence, microscopic satellitosis present in the re‐excision specimen, in‐transit, lymph node, or distant metastasis, or the occurrence of unresectable, locally advanced disease.

Control cases of BAP1‐inactivated melanomas were obtained similarly, but required both loss of the *BAP1* locus on 3p and loss of BAP1 nuclear expression by IHC. In order to be classified as melanoma, an adverse event must have occurred, or the histologic features were fully diagnostic of melanoma in the absence of copy number data or clinical follow‐up.

### SNP microarray testing

To perform SNP microarray testing, we employed the OncoScan FFPE Express 3.0 assay kit (Affymetrix, Santa Clara, CA, USA) per the manufacturer's recommendation. In brief, for each tumour, 10 unstained FFPE sections cut at 10 μm were obtained, each was macrodissected using a haematoxylin and eosin (H&E)‐stained section as a guide, and DNA was extracted using the QIAmp DNA FFPE Tissue Kit (Qiagen, Dusseldorf, Germany) per the manufacturer's protocols. For each lesion a total of 80 ng of DNA was annealed to a molecular inversion probe (MIP) panel, and annealed MIPs were amplified and hybridized to microarrays, as previously described.^2^ The microarrays were analysed and interpreted using OncoScan Console and Nexus Express for OncoScan 3 software (BioDiscovery, El Segundo, CA, USA) or Chromosome Analysis Suite v4.4.0.63 (ChAS; Applied Biosystems, Waltham, MA, USA). CNAs less than 3 MB in size were excluded.

### Morphologic assessment

For each case, the morphology was assessed by a board‐certified dermatopathologist (S.C.B.), and the following features were recorded: depth of dermal involvement (Breslow depth for melanomas), mitoses per mm^2^, deep mitoses (present at a depth of ≥1 mm), ulceration, symmetry, maturation in the BAP1‐inactivated component, the presence of a junctional component, expansile or sheet‐like growth, infiltrative growth (defined as the presence of single cells ramifying among dermal collagen fibres), residual or adjacent nevus, and inflammatory response (absent, nonbrisk, or brisk). In addition, the cytomorphologic features of the BAP1‐inactivated component were classified as epithelioid, plasmacytoid, rhabdoid (epithelioid cells with eosinophilic paranuclear intracytoplasmic inclusions), nevoid (small round cells), or deep penetrating nevus (DPN)‐like (ovoid to polygonal cells with pale‐staining/grey or finely pigmented cytoplasm), and the presence or absence of adipocytic metaplasia and nuclear pseudoinclusions were noted.

### Immunohistochemistry

In a subset of cases, IHC was performed for Ki67, BAP1, BRAFV600E, HMB‐45, β‐catenin, or p16 by the primary dermatopathologist; these studies were reviewed, and the expression levels recorded. For BAP1 IHC, sections of 4‐μm thickness were deparaffinized and heat‐induced epitope retrieval was performed on the Ventana Benchmark Ultra immunostainer using cell conditioning 1 (CC1) buffer from Ventana Medical Systems (Tucson, AZ, USA). After blocking endogenous peroxidase activity, the slides were incubated with a Bap1 antibody (clone C‐4, Santa Cruz Biotechnology, Santa Cruz, CA, USA) for 32 min at 37C. Immunoreactivity was detected by using the OptiView universal DAB detection kit (Ventana Medical Systems).

### Statistical analysis

The reported *P* values were determined using Fisher's exact test applied to 2 × 2 contingency tables (GraphPad Prism 10, San Diego, CA, USA). Cutoffs of ≥3 mitoses/mm^2^ and ≥6 CNAs were used.

## Results

A total of 19 BIMs met the inclusion criteria. To determine if histologic differences were detectable with increasing CNAs, the 19 BIMs were divided into those with ≥2 CNAs (15 cases) and those without any CNAs beyond 3p deletion (0–1 CNAs; four cases). An additional five BAP1‐inactivated melanomas were identified and included as controls.

Overall, the 24 patients ranged in age from 16 to 77 years, with a mean age of 50 years, and included 14 males and 10 females (Table 1). Those with ≥2 CNAs had an average age of 52 years, while those with 0–1 CNA averaged 39 years. The patients with BAP1‐inactivated melanomas averaged 54 years. Out of all cases, 18/24 were encountered in routine practice at the University of Michigan and the remainder were outside consultations. For the 13/19 lesions with available clinical history, the reasons for biopsy included pain (5/13), colour change (4/13), recent growth (3/13), and/or pruritus (2/13). For 15 of 19 lesions with available gross descriptions, the clinical presentation was a round, pink to brown papule ranging from 4 to 22 mm (mean 7.5 mm with a standard error of the mean [SEM] of 1.2 mm). The lesions were located on the head and neck (11/19), extremity (5/19), and trunk (3/19).

**Table 1:** Clinical characteristics of BAP1‐inactived melanocytic tumours

Case	Age	Sex	Anatomic site	Treatment	SLN biopsy	Germline status	Follow‐up (mo.)	Adverse outcome
Tumours with ≥2 CNAs								
1	67	M	Right upper back	Re‐excision with 0.5 cm margins	NP	Clinically suspected positive, declined testing^a^	20	None
2	19	F	Left shoulder	Re‐excision with 2.0 cm margins	—	Clinically suspected positive, declined testing^b^	89	None
3	64	F	Left upper eyelid	Re‐excision	NP	NA	57	None
4	61	F	Right forearm	Re‐excision	—	NA	37	None
5	68	M	Left shoulder	Re‐excision	—	Clinically low risk, no significant personal or family history	40	None
6	54	M	Right superior helix	Re‐excision with 0.5 cm margins	NP	Clinically suspected positive, declined testing^c^	94	None
7	23	F	Right parietal scalp	Re‐excision	NP	NA	57	None
8	40	M	Right ear helix	Re‐excision with 1.0 cm margins	—	NA	27	None
9	74	M	Right lower back	No documented further treatment (excisional biopsy with negative margins)	NP	NA	22	None; died of metastatic lung adenocarcinoma
10	62	F	Left occipital scalp	Re‐excision with 1.5 cm margins	—	Clinically low risk, no significant personal or family history	5	None
11	17	F	Left superior cheek	Re‐excision with 1.0 cm margins	—	Confirmed negative	8	None
12	41	M	Left superior helix	Re‐excision with 0.3 cm margins	NP	Clinically suspected positive, declined testing^d^	19	None
13	58	F	Left buttock	Re‐excision with 0.5 cm margins	NP	NA	41	None
14	76	M	Left anterior proximal thigh	No documented further treatment (punch biopsy with negative margins)	NP	NA	10	None
15	55	M	Left anterior earlobe	Re‐excision with 0.5 cm margins	NP	NA	63	None
Tumours with 0–1 CNA								
16	63	M	Left clavicular neck	Re‐excision with 0.4 cm margins	NP	NA	10	None
17	43	M	Left posterior shoulder	Re‐excision with 1.0 cm margins	NP	Confirmed negative	NA	NA
18	16	M	Left superior lateral neck	Re‐excision	NP	NA	61	None
19	33	M	Right chin	Re‐excision with 0.5 cm margins	NP	NA	26	None
Melanomas								
1M	77	F	left medial plantar foot,^e^ left medial ankle,^f^ and left medial malleolus^f^	Pembrolizumab and talimogene laherparepvec (complete response)	NP	Clinically low risk, no significant personal or family history	25	Presented with multiple in‐transit and satellite metastases
2M	71	F	Right wrist	Re‐excision with 2.0 cm margins	—	NA	3	Re‐excision showed microscopic satellitosis
3M	39	F	Suprapubic	Re‐excision with 1.0 cm margins	—	NA	46	None
4M	32	M	Right lateral malar cheek	Re‐excision with 2.0 cm margins Ipilimumab/nivolumab with complete response	—	NA	79	Regional lymph node metastasis, distant metastases (liver, bone, spleen)
5M	51	M	Right lateral thigh	NA	NA	NA	NA	NA

CR, complete response; NA, not available; NP, not performed.

^a^ The patient had a history of cutaneous melanoma with BAP1 deletion and a family history of cutaneous melanoma.

^b^ The patient had a history of cutaneous melanoma, a family history of cutaneous and uveal melanoma, and had developed four additional BAP1‐inactivated nevi confirmed by IHC.

^c^ The patient had a family history of cutaneous melanoma, uveal melanoma, renal cell carcinoma, and BIMTs.

^d^ The patient hae a history of cutaneous melanoma and had developed numerous biopsy‐confirmed BAP1‐inactivated nevi.

^e^ Presumed primary tumour.

^f^ Presumed in‐transit metastases.

In 13 of 19 patients with BIMs, a detailed personal and family history was available; of these, four patients (31%) were thought to be at high risk for a germline *BAP1* mutation (Table 1). The first such patient had a personal history of BAP1‐inactivated cutaneous melanoma, as well as a family history of early onset melanoma. The second patient had a personal history of cutaneous melanoma, a family history of cutaneous and uveal melanoma, and over 7 years of follow‐up, developed four additional BAP1‐inactivated melanocytic tumours (BIMTs) confirmed by IHC. The third patient had a family history of cutaneous melanoma, uveal melanoma, renal cell carcinoma, and BIMTs. The fourth patient had a personal history of melanoma and numerous biopsy‐confirmed BIMTs. All four of these patients were offered but declined germline *BAP1* testing. The remaining 9/13 patients had no significant personal or family history of malignancy.

**Figure 1:**
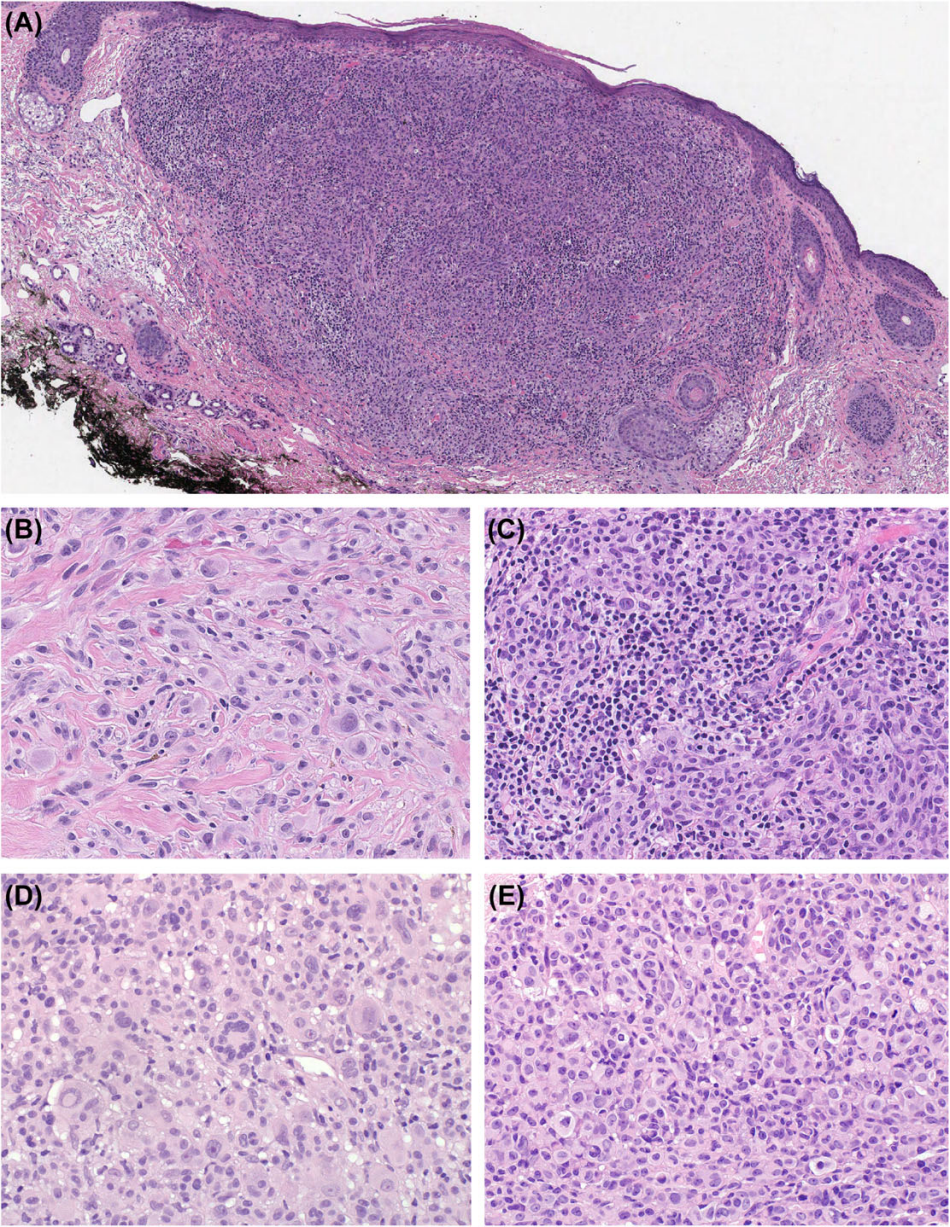
Typical features of BAP1‐inactivated melanocytomas. (A) Case 3. Scanning magnification image showing a dome‐shaped predominantly intradermal melanocytic proliferation with an associated patchy lymphoid infiltrate (H&E, original magnification ×80). (B–E) Cases 1, 3, 7, and 10, respectively. Close inspection reveals epithelioid melanocytes with irregular, pleomorphic nuclei, abundant palely eosinophilic cytoplasm, variable intranuclear inclusions, and distinct cell borders (H&E, original magnifications ×400). [Color figure can be viewed at wileyonlinelibrary.com]

**Figure 2:**
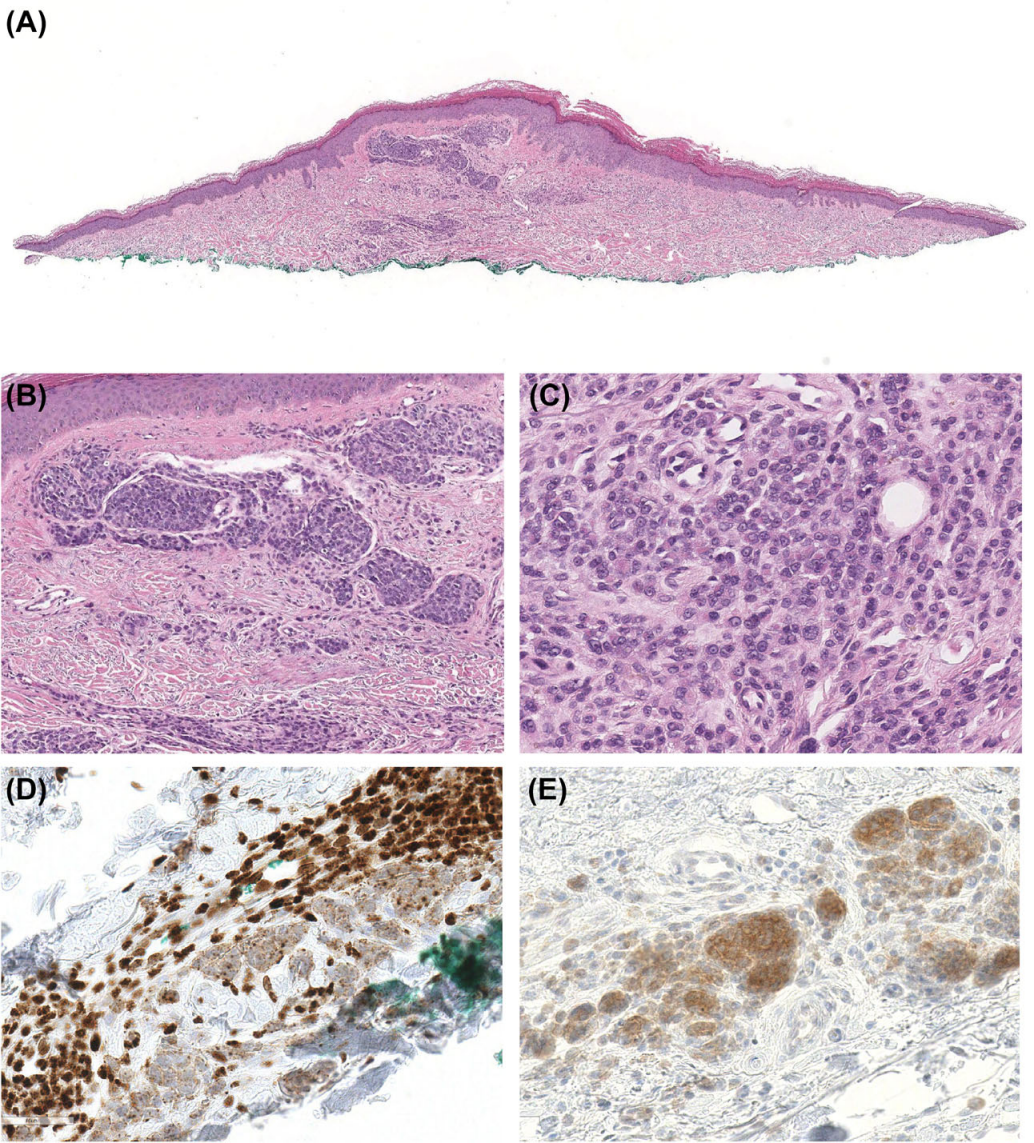
Case 4. (A) Scanning magnification image showing an asymmetric predominantly intradermal melanocytic proliferation that is nested superficially and disperses with dermal descent. (H&E, original magnification ×20). (B) Higher magnification reveals that the composite melanocytes are arranged in large, expansile nests and as single cells (H&E, original magnification ×100). (C) Cytologically, most tumour cells are small, with less cytoplasm that is typically observed in BAP1‐inactived melanocytomas. Many of the cells contain rhabdoid intracytoplasmic inclusions that peripherally displace the nuclei. Scattered smaller nevomelanocytes are seen in the background (H&E, original magnification ×200). (D) BAP1 immunohistochemistry (IHC) revealing a lack of BAP1 nuclear expression in larger melanocytes with retention of expression in background nevus (original magnification ×400). (E) BRAFV600E IHC showing diffuse cytoplasmic expression (original magnification ×400). [Color figure can be viewed at wileyonlinelibrary.com]

**Figure 3:**
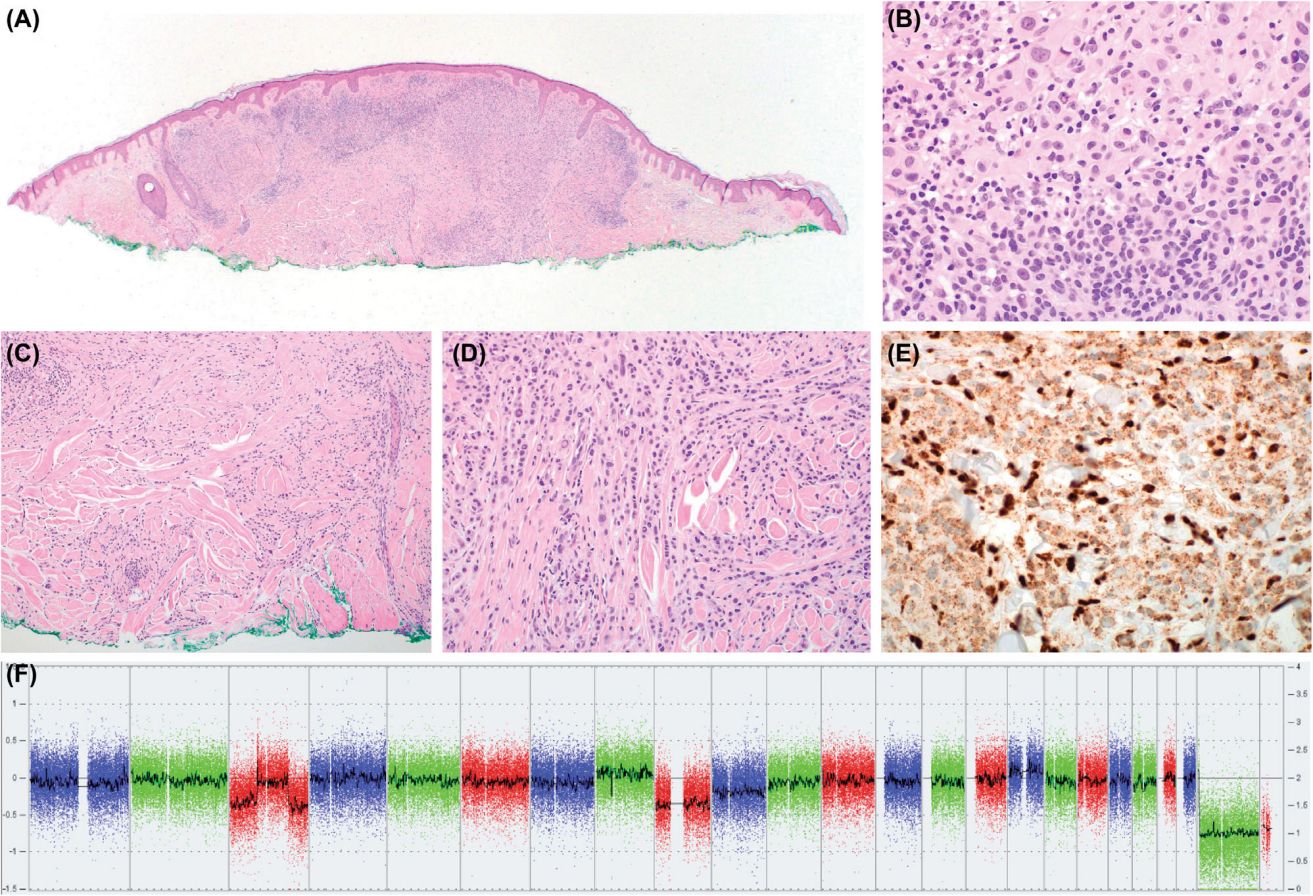
Case 5. (A) Scanning magnification image showing an asymmetric predominantly intradermal melanocytic proliferation. A patchy lymphocytic infiltrate is also present. (B) The composite melanocytes consist of large, atypical epithelioid forms as well as smaller nevoid melanocytes that demonstrate variable cytologic atypia. (C,D) Prominent infiltrative and single‐file growth is seen at the base of the lesion. Although the majority of cells are small in size in the deep dermis, occasional large, epithelioid forms are also visualized. (H&E, original magnifications ×100 and ×200). (E) BAP1 immunohistochemistry demonstrates loss of nuclear expression with punctate cytoplasmic staining throughout the lesion and retention in infiltrating inflammatory cells (H&E, original magnification ×400). (F) SNP microarray analysis showing multiple abnormalities, including part of the 3p arm (3p26.3 – p14.1) containing the *BAP1* locus, part of 3q, entire chromosome 9 (but without *CDKN2A/CDKN2B* homozygous loss), entire chromosome 10 (subset, ~10% of cells), and gain of entire chromosome 16 (subset, ~10% of cells). [Color figure can be viewed at wileyonlinelibrary.com]

**Figure 4:**
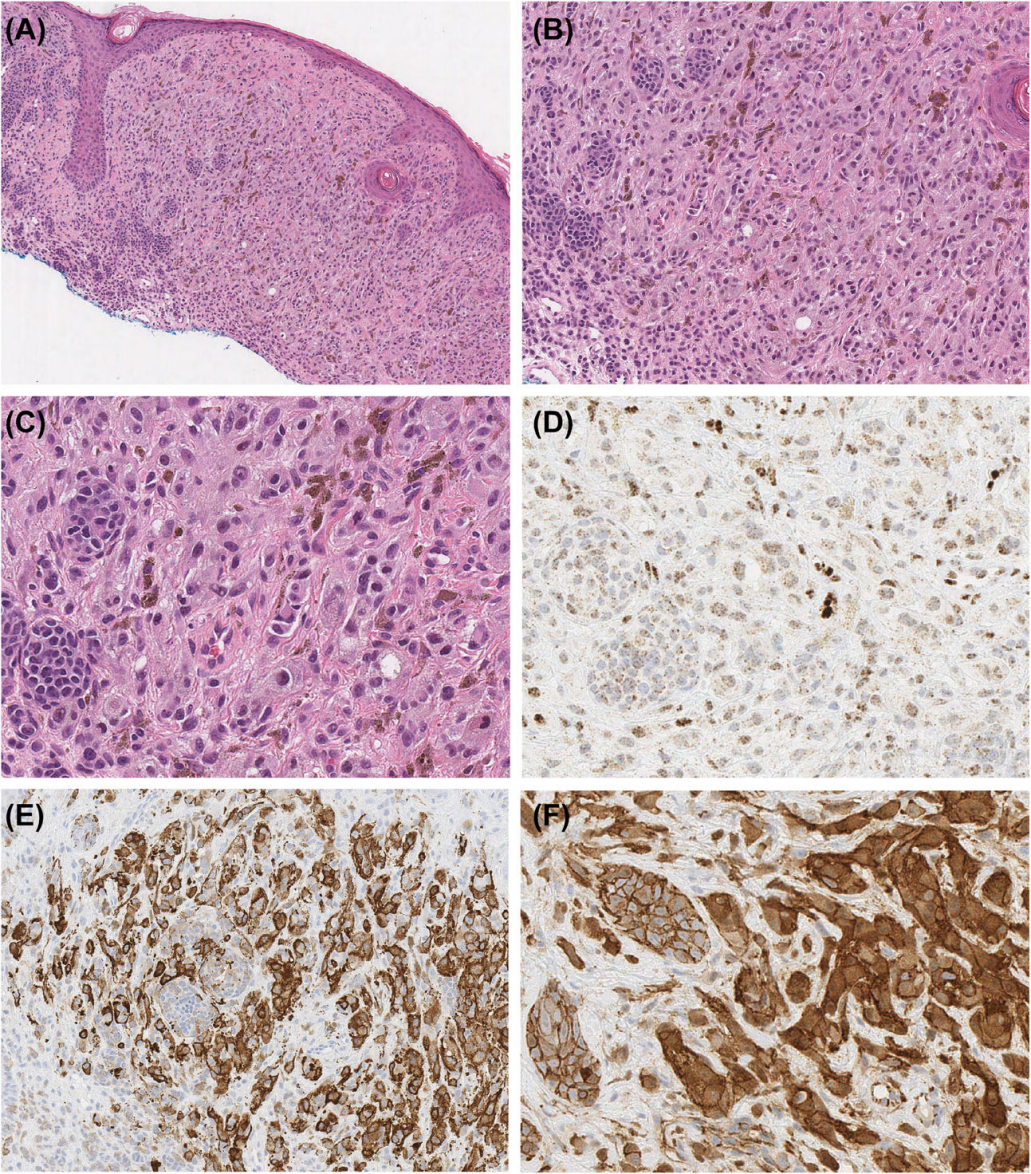
Case 8. (A) Low‐magnification image showing a biphenotypic and asymmetric intradermal proliferation of melanocytes (H&E, original magnification ×40). (B) Closer inspection reveals that the majority of the cells are arranged in sheets and show abundant grey cytoplasm with numerous associated melanophages. Smaller cells present in tight nests and resembling ordinary nevus are also present (H&E, original magnification ×200). (C) High magnification reveals vesicular nuclei with variably prominent nucleoli in the epithelioid population. (H&E, original magnification ×400). (D) BAP1 immunohistochemistry (IHC) showing loss of expression in both populations (original magnification ×400). (E) HMB‐45 IHC showing strong and diffuse staining in the epithelioid component, which is absent in cells resembling ordinary nevus (original magnification ×200). (F) β‐catenin IHC showing strong cytoplasmic and nuclear expression in the larger melanocytes with membranous expression in cells resembling ordinary nevus (H&E, original magnification ×400). [Color figure can be viewed at wileyonlinelibrary.com]

On histologic examination, all 15 cases with ≥2 CNAs showed a nodular or dome‐shaped lesion (Figures 1, 2, 3, 4); the architecture was symmetric in 10/15 cases (Figure 1A), with definitive background BAP1‐retained nevus present in 7/15 (Figure 2B) and not identified in 8/15 cases (Table 2). The depth of dermal involvement ranged from 1.0 to 11 mm, with a mean of 2.5 mm and SEM of 0.6 mm. No lesions displayed ulceration. The median mitotic rate was 1/mm^2^ (range 0–6/mm^2^). Deep mitoses and a mitotic rate of 2 or more per mm^2^ were present in 5/15 and 4/15 cases, respectively. All lesions exhibited expansile or sheet‐like growth (Figures 1, 2, 3, 4). In 4/15 lesions there was an extensive junctional component, while this was focal in 5/15 and absent in 6/15 lesions. Maturation was absent in 5/15 cases, and present but incomplete in 9/15 cases (Figure 3B–D), and the remaining case showed maturation in the nevoid component but none in a deep penetrating nevus‐like subpopulation. Infiltrative growth was present in 7/15 (Figure 3C,D), absent in 5/15, and not evaluable in 3/15 cases due to transection. An inflammatory response was brisk/diffuse in 3/15 (Figure 1A,C), nonbrisk/patchy and associated with the BAP1‐inactivated component in 7/15 (Figures 1B and 3B), and absent in 5/15 cases.

**Table 2:** Histopathologic and immunophenotypic features of BAP1‐inactived melanocytic tumours

Case	Depth (mm)	Mitoses/mm2	Deep mitoses	Ki67 proliferation in BAP1‐inactivated component (%), maximum	Ulceration	Junctional component	Profile	Cytology of atypical BAP1‐inactivated component	Maturation	Inflammatory response	Adipocytic metaplasia	Intranuclear pseudoinclusions	Background nevus	Infiltrative growth	Expansile or sheet‐like growth	BAP1 IHC	BRAFV600E	p16 IHC	HMB‐45 IHC
Tumours with ≥2 CNAs																			
1	1.4	0	−	NP	−	+, limited	Symmetric	Epithelioid	Yes, incomplete	Present, non‐brisk	Absent	Present	Present	+	+	Lost in epithelioid component, retained in background nevus	+ (SNP array)	NP	NP
2	2.0	1	+	5	−	+, extensive	Asymmetric	Epithelioid and plasmacytoid	Yes, incomplete	Absent	Absent	Present	Present	+, Multifocal	+	Lost in epithelioid and plasmacytoid component; retained in background nevus	+ (SNP array)	+	Stratified
3	1.1	1	+	2–3	−	+, limited	Symmetric	Epithelioid	No	Present, brisk	Absent	Absent	Absent	−	+	Lost throughout	+ (SNP array)	+	Scattered cells
4	≥1.0	0	−	1	−	−	Asymmetric	Epithelioid and rhabdoid	Yes, incomplete	Absent	Absent	Focally present	Present	+, Multifocal	+	Lost in epithelioid and rhabdoid component; retained in background nevus	+ (IHC)	NP	NP
5	≥2.2	2	−	3–5	−	−	Asymmetric	Epithelioid and nevoid	Yes, incomplete	Present, non‐brisk	Absent	Present	No definitive	+	+	Lost throughout	+ (IHC)	−	Scattered cells
6	3.3	1	−	NP	−	−	Symmetric	Epithelioid and nevoid	No	Absent	Absent	Present	No definitive	−	+	Lost throughout	NP	NP	Scattered cells
7	2.7	6	+	5–10	−	+, extensive	Symmetric	Epithelioid and nevoid	No	Present, brisk	Absent	Present	Present (special site features)	+, focal	+	Lost in epithelioid component, retained in background nevus	+ (IHC)	+	Scattered cells
8	≥1.0	1	−	5–10	−	+, limited	Asymmetric	DPN‐like (nuclear β‐catenin +) and nevoid	Absent in DPN‐like component, present nevoid component	Present, non‐brisk	Absent	Present	No definitive	NE	+	Lost throughout	−^a^	+	Strong and diffuse in DPN‐like component, negative in nevoid component
9	11	4	+	7–10	−	−	Symmetric	Epithelioid, rhabdoid, plasmacytoid, and nevoid	No	Present, non‐brisk	Absent	Focally present	No definitive	+, focal	+	Lost throughout	+ (IHC)	+	Scattered cells
10	≥1.8	1	−	5–10	−	−	Symmetric	Epithelioid	No	Present, non‐brisk	Absent	Present	Absent	NE	+	Lost throughout	+ (SNP array)	+	−
11	2.5	2	+	5–10	−	+, extensive	Symmetric	Epithelioid, rhabdoid, and plasmacytoid	Yes, incomplete	Present, brisk	Absent	Absent	Present	−	+	Lost in epithelioid, rhabdoid, and plasmacytoid component; retained in background nevus	+ (SNP array)	+	Stratified
12	1.9	1	−	1	−	+, limited	Symmetric	Epithelioid and nevoid	Yes, incomplete	Present, non‐brisk	Absent	Present	No definitive	NE	+	Lost throughout	−	+	Scattered cells
13	1.1	0	−	2–3	−	+, extensive	Symmetric	Epithelioid and nevoid	Yes, incomplete	Absent	Absent	Absent	Present	−	+	Subset loss in epithelioid and nevoid component; retained in background nevus	+ (IHC)	+	NP
14	1.1	0	−	1–2	−	−	Symmetric	Epithelioid and nevoid	Yes, incomplete	Absent	Absent	Focally present	Present	+	+	Lost in epithelioid and nevoid components, retained in background nevus	− (IHC)	− (majority)	−
15	3.8	0	−	2–3	−	+, limited	Asymmetric	Epithelioid, rhabdoid, and nevoid	Yes, incomplete	Present, non‐brisk	Present	Present	No definitive	−	+	Lost throughout	+ (IHC)	− (majority)	Scattered cells
Tumours with 0–1 CNA																			
16	1.0	0	−	2–3	−	−	Asymmetric	Epithelioid, rhabdoid, and nevoid	No	Present, non‐brisk	Absent	Focally present	Present	−	+	Lost in epithelioid, rhabdoid, and nevoid component; retained in background nevus	+ (IHC)	+	Scattered cells
17	0.9	1	−	1–2	−	+, limited	Asymmetric	Epithelioid and nevoid	Yes, incomplete	Absent	Absent	Focally present	Present	+	−	Loss in epithelioid and nevoid component; retained in background nevus	− (IHC)	+	Scattered cells
18	1.8	0	−	NP	−	+, limited	Symmetric	Epithelioid	No	Present, non‐brisk	Absent	Focally present	No definitive	NE	−	Retained	+ (SNP array)	+	Stratified
19	1.5	0	−	3–5	−	−	Asymmetric	Epithelioid and nevoid	Yes, incomplete	Present, non‐brisk	Absent	Present	Present	NE	+	Lost in epithelioid and nevoid component; retained in background nevus	NP	+	Scattered cells
Melanomas																			
1M	2.3	1	+	5–10	−	+, limited	Asymmetric	Moderately sized/monotonous (melanoma); epithelioid and nevoid (background BIM)	No	Present, non‐brisk	Absent	Focally present	No definitive	NE	+	Lost throughout	−^b^ (NGS)	+	−
2M	5.2	3	+	5–10	−	−	Asymmetric	Epithelioid, rhabdoid, spindled, nevoid	Yes, incomplete	Present, non‐brisk	Absent	Present	No definitive	+	+	Lost throughout	+ (IHC)	+	NP
3M	1.5	2	+	3	−	+, extensive	Symmetric	Epithelioid to spindled and heavily pigmented	Yes, incomplete	Present, brisk	Absent	Absent	No definitive	−	+	Lost in epithelioid and spindled heavily pigmented component, retained in deeper nevoid melanocytes	+ (IHC)	+	NP
4M	1.9	3	−	5	−	+, extensive	Asymmetric	Epithelioid and nevoid	No	Present, non‐brisk	Absent	Focally present	No definitive	+	+	Subset loss in epithelioid and nevoid components	+ (SNP array)	+	Strong, diffuse
5M	4.2	4	+	5–10	−	+, extensive	Asymmetric	Epithelioid with uniformly enlarged nuclei and prominent nucleoli (melanoma); epithelioid, rhabdoid, and nevoid (background BIM)	Yes, incomplete in background BIM; no in melanoma	Present, non‐brisk	Present in background BIM; absent in melanoma	Absent	No definitive	− (background BIM); + (melanoma)	+ (background BIM and melanoma)	Lost throughout	+ (IHC)	+	NP

BIM, BAP1‐inactivated melanocytoma; DPN, deep penetrating nevus; IHC, immunohistochemistry; NE, not evaluable (e.g. due to transected sample); NGS, next‐generation sequencing; NP, not performed.

^a^ This lesion was positive for an *NRAS* mutation. Beta catenin was positive in a deep penetrating nevus‐like component.

^b^ Positive for *NRAS* Q61R mutation.

In most cases (14/15), the BAP1‐inactivated melanocytes included epithelioid cells with abundant palely eosinophilic cytoplasm, large vesicular nuclei, and prominent nucleoli (Figure 1B–E). A minority of those cases also displayed intermixed cells with plasmacytoid (3/15) and/or rhabdoid (4/15; Figure 2C) cytomorphology. The remaining case (case 8) displayed DPN‐like cells as well as conventional nevus‐like cells (Figure 4A–C). Intranuclear pseudoinclusions were present diffusely in 9/15 cases (Figure 1D) and focally in 3/15 cases. One case displayed adipocytic metaplasia.

BAP1 IHC showed loss of nuclear reactivity in at least a subpopulation of cells in all 15 cases with ≥2 CNAs (Figures 2, 3, 4). The overall Ki67 proliferation index was ≥5% in 7 of 13 cases for which this was available. Of the 14 lesions for which BRAFV600E testing was performed, 11 were positive (six by IHC and five by microarray; Figure 2E). Expression of p16 by IHC was retained in 9 of 12 cases for which this study was available. HMB‐45 expression was stratified (strong expression in superficial dermal melanocytes with diminution of signal with dermal descent; 2/12 cases), seen only in scattered cells (7/12), or absent (2/12) in 11/12 cases in which it was performed (Table 2). One case (case 8) showed strong and diffuse expression of HMB‐45 in DPN‐like cells (Figure 4E) that were also positive for nuclear β‐catenin (Figure 4F).

SNP microarray analysis in cases with ≥2 CNAs identified copy number losses including 3p21 that ranged in size from 0.22 Mb to the loss of entire chromosome 3 (Table 3). Homozygous loss of 3p21 was seen in one case. By definition of this subgroup, all 15 cases showed multiple copy number abnormalities (e.g. Figure 3F); the median was three (range 2–6). Beyond deletion of 3p, the most common additional copy number loss involved heterozygous loss of 9p21 (7/15 lesions) spanning the *CDKN2A* locus. Other abnormalities included one case (1/15) with copy number gain involving the *MYC* locus at 8q24 and one case (1/15) with a deletion of 17q spanning the *BRCA1* locus at 17q21. There was one case displaying gain of 6p25 (1/15) and no cases showing gain of 11q13 or loss of 6q23, which involve the *RREB1*, *CCND1*, and *MYB* loci, respectively.

**Table 3:** SNP microarray aberrations in BAP1‐inactivated melanocytic tumours

Case	Total CNAs	Cytoband (start – end)	Type	Genomic coordinates (start – end)	Size (Mb)	CNAs involving important oncogenes or tumour suppressors
Tumours with ≥2 CNAs						
1	5	3p22.1 – p14.3 5p15.33 – p11 5q11.1 – q35.3 9p24.3 – q34.3 20q11.21 – q13.33	Loss Gain Loss Loss Gain	39,472,525 – 55,281,622 1–46,401,271 49,331,260 – 180,813,336 1–141,054,761 29,471,088 – 62,918,998	15.81 46.40 131.48 141.05 33.45	heterozygous loss of *CDKN2A* (9p21)
2	2	3p26.3 – p14.2 9p24.3 – q31.3	Loss Loss	63–62,729,934 184–114,033,877	62.67 113.85	heterozygous loss of *CDKN2A* (9p21)
3	4	3p26.3 – q29 4p16.3 – q35.2 9p24.3 – p11.2 Xp22.33 – q28	Loss Gain Loss Loss	1–198,022,430 1–191,154,276 205–44,893,094 1–155,270,560	198.02 191.15 44.69 155.27	heterozygous loss of *CDKN2A* (9p21)
4	6	2p23.3 – p23.3 3p26.3 – p14.3 11p11.2 – p11.2 11q12.1 – q12.1 17p13.3 – p11.2 17q12 – q21.33	Loss Loss Loss Loss Loss Loss	26,110,142 – 27,231,145 63,410 – 57,528,503 45,869,657 – 47,834,346 57,443,841 – 58,319,045 400,958 – 17,078,849 36,760,365 – 48,993,880	1.12 57.47 1.96 0.88 16.68 12.23	Deletion of *BRCA1* (17q21)
5	5	3p26.3 – p14.1 3q24 – q29 9p24.3 – q34.3 10p15.3 – q26.3 16p13.3 – q24.3	Loss Loss Loss Loss Gain	63,410 – 69,147,862 147,745,544 – 197,852,564 204,737 – 141,054,761 126,069 – 135,434,303 83,886 – 90,158,005	69.08 50.11 140.85 135.31 90.07	heterozygous loss of *CDKN2A* (9p21)
6	2	1q23.1 – q23.1 3p26.3 – p12.1	Loss Loss	156,805,697 – 156,909,695 63,411 – 83,534,398	0.10 83.47	
7	2	3p22.2 – p14.3 Xp22.33 – q28	Loss Loss	37,258,063 – 56,561,883 178–155,219,364	19.30 155.04	
8	6	3p21.1 – p21.1 6p25.3 – q27 7p22.3 – q36.3 9p24.3 – q34.3 12q11 – q15 20p13 – q13.33	Loss Gain Gain Loss Loss Gain	52,047,420 – 52,589,840 204,908 – 170,913,051 2,264,393 – 159,118,443 204,737 – 141,054,761 37,902,897 – 70,403,306 69,093 – 62,912,463	0.54 170.71 156.65 140.85 32.50 62.84	Gain of *RREB1* (6p25) heterozygous loss of *CDKN2A* (9p21)
9	4	3p22.2 – p21.1 3p21.1 – p21.1 6q13.13 – q13.33 20q13.13 – q13.33	Loss HMZ loss Loss Gain	37,957,785 – 52305,633 52,322,797 – 52,536,308 93,992,783 – 171,018,664 47,260,263 – 62,912,463	14.35 0.21 77.03 15.65	
10	3	3p21.1 – p21.1 8q23.3 – q24.3 15q11.1 – q15.2	Loss Gain Gain	52,389,666 – 52,610,415 113,441,607 – 142,703,241 20,161,372 – 43,175,251	0.22 29.26 23.01	Gain of *MYC* (8q24)
11	2	3p26.3 – p14.1 3q12.1 – q29	Loss Loss	63,410 – 67,672,171 99,326,101 – 197,852,564	67.61 98.53	
12	3	3p26.3 – q29 9p24.3 – q34.3 10p25.3 – q26.3	Loss Loss Loss	63,410 – 197,852,564 204,737 – 141,054,761 126,069 – 135,434,303	197.79 140.85 135.31	heterozygous loss of *CDKN2A* (9p21)
13	2	3p26.3 – q29 Xp22.33 – q28	Loss Loss	1–198,022,430 1–155,270,560	198.02 155.27	
14	2	3p26.3 – q29 9p24.3 – q34.3	Loss Loss	63,410 – 197,852,564 204,737 – 140,876,480	197.79 140.67	heterozygous loss of *CDKN2A* (9p21)
15	2	3p26.3 – q29 11p15.5 – p11.12	Loss Loss	63–197,852,564 193–51,575,951	197.79 51.38	
Tumours with 0–1 CNA						
16	0	‐	‐	‐	‐	
17	1	3p24.3 – p21.1	Loss	18,735,850 – 53,007,995	34.27	
18	1	3p26.3 – q29	Loss	63–197,852,564	197.97	
19	1	3p26.3 – q29	Loss	63–197,858,436	197.80	
Melanomas						
1M	6	3p26.3 – q29 8p23.3 – p11.1 8q11.1 – q24.3 16q22.1 – q24.3 21p11.2 – q22.3 Xp22.33 – q28	Loss Loss Gain Loss Loss Loss	63,410 – 197,852,564 172,416 – 43,767,534 46,896,971 – 146,292,734 69,967,731 – 90,158,005 9,648,314 – 48,097,610 177,941 – 155,219,364	197.79 43.60 99.40 20.19 38.45 155.04	Gain of *MYC* (8q24)
2M	6	1p36.31 – p13.3 3p26.3 – q29 6p25.3 – p22.3 6q11.1 – q27 9p24.3 – q34.3 14q11.2 – q32.33	Loss Loss Gain Loss Loss Loss	7,015,632 – 109,070,032 63,410 – 197,852,564 204,908 – 13,009,496 61,886,392 – 170,913,051 204,737 – 107,282,024 20,219,082 – 107,282,024	102.05 197.79 22.80 109.03 87.06 87.06	Gain of *RREB1* (6p24) Loss of *MYB* (6q23)
3M	10	1p36.33 – p13.2 3p26.3 – q29 9p24.3 – q31.1 10p15.3 – q23.31 10q23.31 – q26.3 10q23.31 – q26.3 12p13.33 – p11.1 12q11 – q24.33 13q11 – q34 20q13.32	Loss Loss Loss Loss Hmz loss Loss Gain Loss Gain Gain	140,423 – 115,320,054 63,411 – 197,852,564 204,738 – 112,867,627 126,070 – 89,634,501 89,635,577 – 89,674,157 89,675,296 – 135,434,303 150,819 – 34,828,211 37,902,988 – 133,818,115 19,011,140 – 115,169,878 56,782,876 – 62,912,463	115.18 197.79 112.66 89.51 0.04 45.76 34.68 95.92 96.16 6.13	Homozygous loss of *PTEN* (10p23)
4M	7	1p36.33 – p12 3p25.1 – p14.1 7p22.1 – p15.2 11p15.5 – q25 17q11.1 – q23.2 17q23.2 – q24.2 17q24.2 – q25.3	Loss Loss Gain Loss Loss Gain Loss	704–118,689,146 13,303,991 – 64,634,029 6,187,883 – 26,441,017 1–135,006,516 25,287,930 – 59,508,677 59,508,678 – 64,350,985 64,350,986 – 80,279,032	117.99 51.33 20.25 135.01 34.22 4.84 15.93	Deletion of *BRCA1* (17q21)
5M	8	1p31.1 – p21.1 3p26.3 – q29 4p16.3 – p14 4p13 – q13.1 9q21.11 – q34.3 10q11.21 – q26.3 12q21.2 – q24.33 17p13.3 – p11.2	Loss Loss Loss Loss Loss Loss Loss Loss	73,204,543 – 105,370,881 63,410 – 197,852,564 69,403 – 37,973,722 41,804,385 – 65,484,033 72,174,527 – 141,054,761 42,413,321 – 135,434,303 78,746,492 – 133,818,115 400,958 – 21,003,053	32.17 197.79 37.90 23.68 68.88 93.02 55.07 20.60	Loss of *TP53* (17p13)

HMZ, homozygous.

Clinical follow‐up was available on all 15 patients in the ≥2 CNA group and ranged from 5 to 94 months with a mean of 39 months. Ten lesions (10/12) were treated with wide local excision with margins ranging from 0.3 to 2 cm; in the remaining two cases, re‐excision was deferred due to comorbidities (case 9) or negative margins on the biopsy specimen (case 14). Sentinel lymph node biopsy (SLNB) was performed for 6/15 cases, with no positive lymph nodes.

All cases containing 0–1 CNAs with available clinical follow‐up (3/3) were not associated with adverse events. On average, these cases had a lower mitotic rate (median 0/mm^2^, range 0–1); none showed deep mitoses. A limited junctional component was seen in 2/4 cases, while a junctional component was not identified in the remaining two cases; 3/4 cases had an asymmetric growth pattern. The depth of dermal involvement ranged from 0.9 to 1.8 mm, with a mean of 1.3 mm and SEM of 0.2 mm. Two cases did not show maturation, while incomplete maturation was seen in the remaining two cases. An accompanying patchy lymphocytic infiltrate was seen in 3/4 cases. Adipocytic metaplasia was not seen in any case. Intranuclear pseudoinclusions were present in all cases (focally in three, diffusely in one). A background nevus was identified in 3/4 cases. Infiltrative growth was seen in 1/2 cases that were not transected. Expansile or sheet‐like growth was seen in 2/4 cases. By IHC, all but one case showed loss of BAP1 expression in a subset of cells, while the remaining case showed retention of nuclear expression, but showed loss of 3p containing the *BAP1* gene by SNP microarray, and otherwise had typical histopathologic features of a BIM. Conversely, one case had no CNAs, including at chromosome 3p, but showed loss of BAP1 expression by IHC. Only one case had a Ki67 proliferation index ≥5%. BRAFV600E was expressed in 2/3 cases in which this assay was performed. Expression of p16 was retained in all cases. HMB‐45 showed a pattern of stratification (1/4) or highlighted scattered cells (3/4) in all cases.

**Figure 5:**
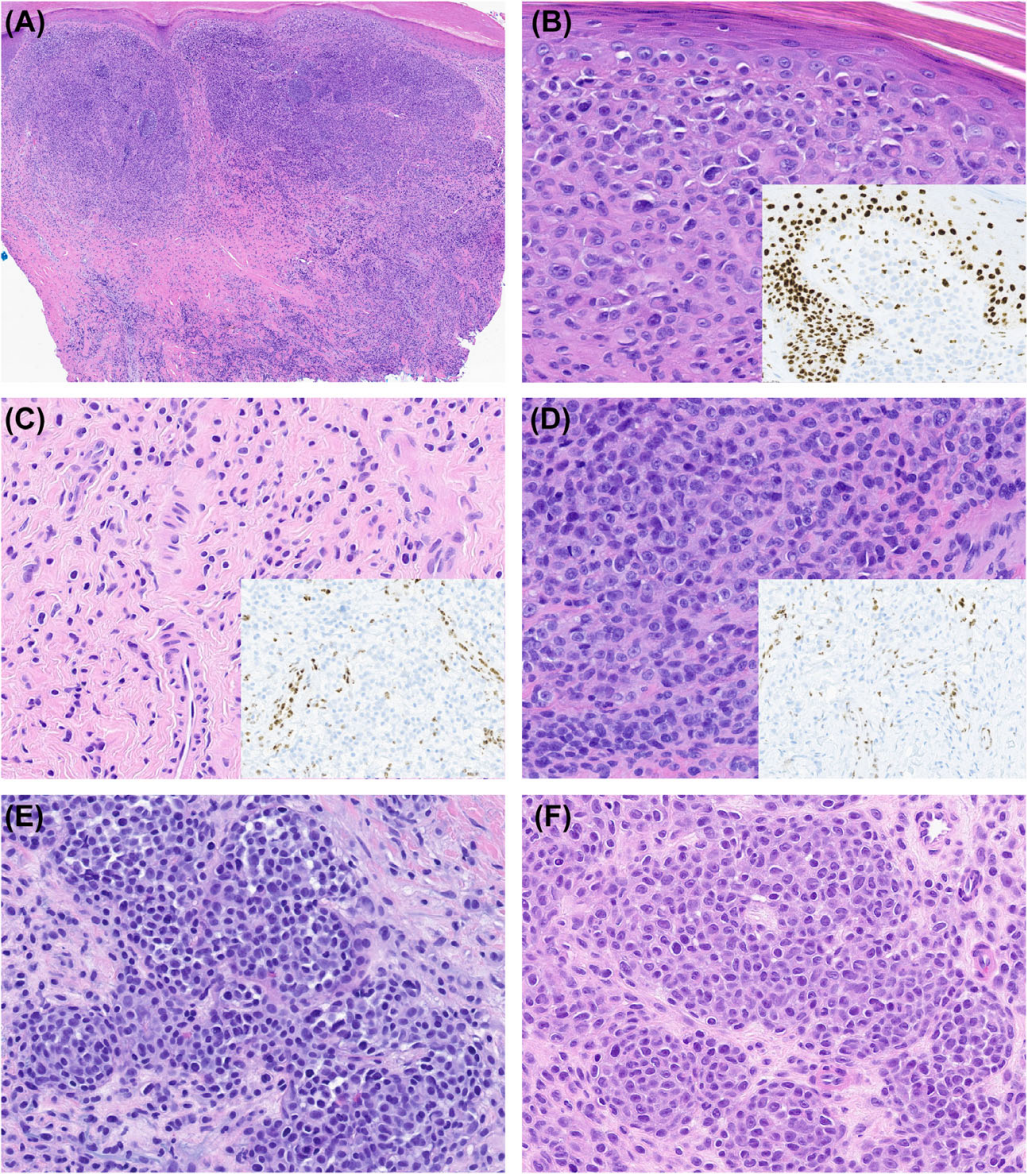
Case 1M. (A) Scanning magnification image of the foot lesion reveals a predominantly intradermal neoplasm with regions of expansile growth superficially and extension into the deep dermis (H&E, original magnification ×46). (B) Higher magnification reveals the presence of a pleomorphic epithelioid component typical of BAP1‐inactivated melanocytomas (H&E, original magnification ×400). BAP1 immunohistochemistry (IHC; inset) showing loss of expression in this component (original magnification ×400). (C) A banal nevoid population (H&E), also BAP1‐inactivated by IHC (inset), is identified in the background (original magnifications ×400). (D) Also within the dermis and comprising the majority of the tumour is a distinct, highly cellular population of cytologically monotonous, moderately sized melanocytes with BAP1 IHC (inset) also showing diffuse loss of expression (original magnifications ×400). (E) Histologic evaluation of the ankle lesion revealed an intradermal population of melanocytes closely resembling the monotonous, moderately sized population in the prior left foot specimen (H&E, original magnification ×400). Expression of BAP1 was diffusely lost. (F) Histologic evaluation of the malleolus specimen also showed a similar population of melanocytes, again closely resembling the prior two specimens with diffuse loss of BAP1 staining (H&E, original magnification ×400). [Color figure can be viewed at wileyonlinelibrary.com]

In contrast to the initial set of 19 BIMs, 3/5 patients with BAP1‐inactivated melanomas experienced an adverse event, and the remaining two cases had histologic features that were fully diagnostic of melanoma. Sentinel lymph node biopsy was negative in 3/3 patients for which this procedure was performed. Of the melanoma cases, one patient (case 1M) presented with locoregionally advanced, unresectable disease, with numerous pink papules and plaques on her left foot and medial ankle. On presentation, lesions on the left medial plantar foot, left medial ankle, and left medial malleolus were biopsied. Histologically, the plantar lesion (Figure 5A) contained three distinct populations of melanocytes: a pleomorphic epithelioid component (Figure 5B), a nevoid population (Figure 5C), and a cytologically monotonous, moderately sized component that comprised the majority of the lesion (Figure 5D). Biopsy specimens of the ankle and malleolus lesions contained only cytologically monotonous, moderately sized melanocytes (Figure 5E,F) and were interpreted as atypical intradermal BIMTs suggestive of locoregional metastases. Overall, the constellation of findings were most consistent with melanoma arising in a BIM. SNP microarray analysis of the primary lesion showed six CNAs including gain of the *MYC* locus at 8q24. Next‐generation sequencing (NGS) revealed an *NRAS* Q61R mutation. SNP microarray of the left medial malleolus tumour showed a nearly identical pattern. Treatment included pembrolizumab (200 mg every 3 weeks for 18 months) and talimogene laherparepvec (T‐VEC, six injections over 3 months, beginning 6 months after starting pembrolizumab). The lesions were noted to decrease in size by at least 50% during treatment with T‐VEC. One month after completing T‐VEC, biopsy specimens of three residual papules on the left ankle showed chronic dermal inflammation and interface dermatitis. SOX10 IHC confirmed no residual melanoma. A positron emission tomography scan showed no metabolic evidence of disease 5 months after completing treatment, and she has developed no new clinical lesions.

Case 5M was similar to case 1M and showed a background melanocytic tumour that showed typical histologic features of a BIM, but with a morphologically distinct clonal population that was higher‐grade, with uniformly enlarged nuclei and prominent nucleoli. This morphologic clone included a highly atypical junctional component that was indistinguishable from typical superficial spreading melanoma *in situ*. Clinical follow‐up was not available for this patient.

The other cases considered to be diagnostic of melanoma showed alternative histologic features. Interestingly, case 2M showed typical morphologic features of BIM without a distinct third clonal outgrowth. The re‐excision specimen showed microscopic satellitosis, confirmed by BAP1 IHC. Personal and family history were unremarkable, rendering the possibility of a separate BIM arising in the context of a *BAP1* germline mutation unlikely. Case 3M did not show typical morphologic features of a BIM despite subset loss of BAP1 expression, but instead was heavily pigmented with epithelioid to spindled melanocytes that exhibited pseudomaturation to nevoid forms in the deeper dermis. Clinical follow‐up was not available for this patient. Lastly, case 4M contained a broad, highly atypical junctional component with pagetoid spread that was indistinguishable from typical superficial spreading melanoma *in situ*. This patient eventually developed regional nodal metastases as well as distant metastases to the liver and spleen but had a complete response to ipilimumab/nivolumab.

Of the five melanomas, the average Breslow depth was 3.0 mm (range 1.5–5.2 mm, SEM 0.7 mm). The median mitotic count was three (range 1–4/mm^2^) compared to one (range 0–6/mm^2^) in the 19 indolent cases. While three of five melanomas had a mitotic rate of ≥3/mm^2^, only 2 of 19 BIMS met this threshold (*P* = 0.04). Other tabulated histologic features other than cytomorphology and the degree of atypia within the junctional component did not significantly differ. Of the melanomas, none were ulcerated, and 3/5 cases showed an extensive junctional component, which was limited in one case and absent in one case; 4/5 cases showed an asymmetric profile on scanning magnification. No maturation was seen in 3/5 cases, while the remaining two cases showed incomplete maturation. A patchy lymphocytic infiltrate was seen in 4/5 cases, with brisk inflammation in the remaining case. Adipocytic metaplasia was seen in an associated typical BIM in one case (case 5M) and was absent in all other cases. Intranuclear pseudoinclusions were at least focally present in 3/5 cases. Expansile or sheet‐like growth was seen in all cases, and infiltrative growth was seen in 3/4 cases that were evaluable for this feature. Lymphovascular or perineural invasion were not directly observed in any case. By IHC, all cases showed at least subset loss of BAP1 expression. A Ki67 proliferation index of ≥5% was observed in 4/5 cases. All but one case was positive for BRAFV600E by either IHC or SNP microarray; the remaining case (case 1M) harboured an *NRAS* Q61R mutation. All cases retained expression of p16. HMB‐45 was performed in two cases, which showed diffuse expression in one case, and was entirely negative in one case.

The median number of CNAs in the BAP1‐inactivated melanomas was seven (range 6–10) compared to two (range 0–6) in indolent cases. While all five cases of melanoma had ≥6 CNAs, only 2/19 BIMs met this threshold (*P* = 0.0005). All examples of melanoma showed at least one aberration involving a major oncogene or tumour suppressor, including one case of each with gain of MYC (8q24), gain of *RREB1* (6p24) in conjunction with loss of *MYB* (6q23), homozygous loss of *PTEN* (10p23), deletion of *BRCA1* (17q21), and loss of *TP53* (17p13).

## Discussion

Here we describe a series of BIMs and BAP1‐inactivated melanomas for which SNP microarray analysis was performed as an ancillary diagnostic test. As previously reported, a typical BIM will consist of background nevus cells and an adjacent population of larger epithelioid cells, the latter often showing sheet‐like or nodular growth and variability in cell size, shape, and nuclear contours.^5^, ^10^ Most of the BIMs we describe have common histopathologic features with those in prior reports.^5^, ^9^, ^10^ Most lesions had at least a limited junctional component (11/19), lacked ulceration (19/19), featured at least a patchy lymphocytic infiltrate (13/19), and had a predominant epithelioid component with expansile or sheet‐like growth (17/19). However, definitive residual conventional nevus was present in only 10/19 (53%) of tumours, fewer than in prior series (e.g. 90% in Donati *et al*.).^9^ Moreover, the mitotic rate in our cases was somewhat higher than in previous reports, with 4/19 (21%) of lesions showing two or more mitoses per mm^2^ and 5/19 (26%) with mitoses near the base of the lesion, compared to 0%–10% and 26% in prior published series, respectively.^5^, ^9^ These differences likely reflect our selection methodology, which favoured difficult‐to‐classify lesions, many from our consultation practice for which SNP microarray analysis was performed based on clinical need.

Cytomorphologically, intranuclear pseudoinclusions in the epithelioid compartment and adipocytic metaplasia are additional histologic features that have been described in BIMs.^9^, ^19^ Conversely, adipocytic metaplasia has been suggested to be lacking in BAP1‐inactivated melanomas, possibly making this a relevant histologic feature.^19^ Interestingly, while most cases in all subgroups showed evidence of intranuclear pseudoinclusions, all cases lacked adipocytic metaplasia except for one melanoma that showed this feature in an associated typical BIM. One tumour in our series lacked the epithelioid morphology characteristic of BIMs, despite showing loss of BAP1 by IHC; this tumour was notable for DPN‐like (with diffuse expression of HMB‐45 and nuclear β‐catenin by IHC) and conventional nevus‐like (yet BAP1‐inactivated) components. Although we cannot entirely exclude nonspecific β‐catenin staining, this case raises the possibility that defects in β‐catenin may supersede BAP1 inactivation to enforce a DPN‐like cytomorphology. To our knowledge, this occurrence (a melanocytoma with both *BAP1* and probable *CTNNB1* alterations) has not been previously reported.

SNP microarray analysis has demonstrated the utility in delineating benign from malignant melanocytic tumours.^4^ Multiple studies have shown that most melanomas (82.5–96.2%) harbour multiple CNAs, while 94.7–100% of benign nevi have none.^26^, ^27^, ^28^, ^29^ Moreover, there is evidence that atypical or indeterminate melanocytic tumours tend to have an intermediate number of CNAs, i.e. between what is typical of benign nevi and melanoma. In a study of 95 melanocytic tumours, Alomari *et al*. found an average number of CNAs of 0 in benign nevi, 0.6 in atypical nevi (range 0–3), 2.8 in intermediate lesions (range 0–17), and 18.1 in melanomas (range 0–61).^2^ In our cohort of BIMs, the median number of CNAs was two, with a range of 0–6. The most common molecular aberration after loss of 3p21 was heterozygous loss of the *CDKN2A* locus, which, unlike homozygous loss, has not been associated with melanoma.^30^, ^31^ Thus far, none of these cases have shown malignant behaviour from available clinical follow‐up. Interestingly, two tumours showed CNAs among the usual loci tested by FISH (6p25, 6q23, Centromere 6, 8q24, and 11q13) for ambiguous melanocytic tumours, suggesting a positive FISH result with these probes. The median number of CNAs (seven) was higher in our set of five melanomas compared to two in all 19 BIMs; however, the ranges overlapped (6–10 for melanomas and 0–6 for BIMs). Furthermore, it is predicted that targeted FISH would have detected copy number aberrations in just three of five melanoma cases.

It has been found that ASTs have a high incidence (39%) of microscopic regional lymph node involvement, but nonetheless have a favourable prognosis.^32^, ^33^ Based on the few available series, BIMs have a much lower incidence of positive SLNB (0% in this cohort), but larger studies will be necessary to determine if BIMs with microscopic lymph node involvement have as favourable a prognosis as ASTs. Nevertheless, as many of the BIMs in this study were reviewed and clinically managed at a time when the biologic potential of these tumours was not well understood, our results support the position that sentinel lymph node biopsy is likely not indicated in most of these tumours, even when multiple CNAs are detected by SNP microarray.

One limitation of this study is that microarray analysis showed homozygous loss involving *BAP1* in only one case, with monoallelic loss of 3p21 in most of the remaining cases. For complete BAP1 inactivation, the other allele would need to acquire an inactivating mutation, insertion, or deletion not detected by SNP microarray. Importantly, BAP1 IHC showed loss of nuclear reactivity in all but one lesion, which showed evidence of copy number loss of 3p by SNP microarray and otherwise had typical histopathologic features of BIM, suggestive of a pathogenic mutation that preserves expression and nuclear localization of the protein, but is catalytically inactivating.^34^ NGS would be valuable to profile inactivating mutations in the *BAP1* gene in these tumours, but was not available. An additional limitation is that genetic testing for germline BAP1 mutations was not performed, as the four patients strongly suspected of germline mutations (out of 13 with accessible personal and family history) declined testing. The rate of 31% (4/13) with likely germline mutations is similar to the 5/9 (56%) previously reported in a similar study.^9^

It has been proposed that BIMs and other melanocytomas occupy a genetically intermediate position between conventional benign nevi and melanoma. Observations supporting this concept include the apparent growth advantage of the BAP1‐inactivated clone over background nevus, the increased risk of cutaneous melanoma in patients with germline BAP1 mutations, and the reported cases of BAP1‐inactivated melanomas.^9^ Nonetheless, most BIMs in the available reported series, have undergone an indolent course, even despite some cases containing genomic aberrations previously associated with melanoma.

Although limited by incomplete follow‐up and a small sample size, our results indicate the need for careful histologic evaluation of BIMTs and integration of all available data to determine a final diagnosis. Although many such lesions are likely to be indolent, rare cases with atypical features, particularly increased proliferative activity and numerous CNAs, should be viewed with some caution. Importantly, the majority of BAP1‐inactivated melanomas (4/5) in this study had histopathologic features well outside of the spectrum of typical BIMs. Additionally, the most common molecular aberration after loss of 3p21 was heterozygous loss of the *CDKN2A* locus, which, unlike homozygous loss, has not been associated with melanoma. However, as such tumours are rare, continued follow‐up and accrual of additional BIMs is needed to fully elucidate the biologic potential of these tumours.

## Author contributions

AAA and SCB designed the study. JSD, NAZ, AAA, and SCB collected the data. JSD, PWH, and SCB drafted the article. All authors (JSD, NAZ, TN, DRF, ACH, LL, RMP, PWH, AAA, and SCB) contributed to reviewing the article and approved the final version.

## Funding information

JSD was supported by NIH grant # T32AR007197.

## Conflict of interest

The authors report no conflicts of interest.

## Data Availability

All deidentified data that were collected are contained in the published article.

## References

[1] Shain AH , Yeh I , Kovalyshyn I *et al*. The genetic evolution of melanoma from precursor lesions. N Engl J Med 2015; 373; 1926–1936. 26559571 10.1056/NEJMoa1502583

[2] Alomari AK , Miedema JR , Carter MD *et al*. DNA copy number changes correlate with clinical behavior in melanocytic neoplasms: proposal of an algorithmic approach. Mod Pathol 2020; 33; 1307–1317. 32066860 10.1038/s41379-020-0499-y

[3] WHO Classification of Tumours Editorial Board . Skin tumours [Internet; beta version ahead of print]. Lyon (France): International Agency for Research on Cancer 2023; (WHO classification of tumours series, 5th ed.; vol. 12). Available from: https://tumourclassification.iarc.who.int/chapters/64.

[4] Andea AA . Molecular testing for melanocytic tumors: a practical update. Histopathology 2022; 80; 150–165. 34958511 10.1111/his.14570

[5] Wiesner T , Murali R , Fried I *et al*. A distinct subset of atypical Spitz tumors is characterized by BRAF mutation and loss of BAP1 expression. Am J Surg Pathol 2012; 36; 818–830. 22367297 10.1097/PAS.0b013e3182498be5 PMC3354018

[6] Wiesner T , Obenauf AC , Murali R *et al*. Germline mutations in BAP1 predispose to melanocytic tumors. Nat Genet 2011; 43; 1018–1021. 21874003 10.1038/ng.910 PMC3328403

[7] Njauw CJ , Kim I , Piris A *et al*. Germline BAP1 inactivation is preferentially associated with metastatic ocular melanoma and cutaneous‐ocular melanoma families. PLoS One 2012; 7; e35295. 22545102 10.1371/journal.pone.0035295 PMC3335872

[8] Murali R , Wilmott JS , Jakrot V *et al*. BAP1 expression in cutaneous melanoma: a pilot study. Pathology 2013; 45; 606–609. 24018818 10.1097/PAT.0b013e3283653818

[9] Donati M , Martinek P , Steiner P *et al*. Novel insights into the BAP1‐inactivated melanocytic tumor. Mod Pathol 2022; 35; 664–675. 34857909 10.1038/s41379-021-00976-7

[10] Yeh I , Mully TW , Wiesner T *et al*. Ambiguous melanocytic tumors with loss of 3p21. Am J Surg Pathol 2014; 38; 1088. 24705312 10.1097/PAS.0000000000000209 PMC4101029

[11] Garfield EM , Walton KE , Quan VL *et al*. Histomorphologic spectrum of germline‐related and sporadic BAP1‐inactivated melanocytic tumors. J Am Acad Dermatol 2018; 79; 525–534. 29753057 10.1016/j.jaad.2018.05.005

[12] Boyd AS , Chen S , Shyr Y . Intradermal nevi with atypical nuclei in the elderly: the senescent nevus. J Am Acad Dermatol 2015; 73; 500–506. 26188628 10.1016/j.jaad.2015.06.013

[13] Zhang AJ , Rush PS , Tsao H , Duncan LM . BRCA1‐associated protein (BAP1)‐inactivated melanocytic tumors. J Cutan Pathol 2019; 46; 965–972. 31233225 10.1111/cup.13530

[14] Masclef L , Ahmed O , Estavoyer B *et al*. Roles and mechanisms of BAP1 deubiquitinase in tumor suppression. Cell Death Differ 2021; 28; 606–625. 33462414 10.1038/s41418-020-00709-4 PMC7862696

[15] Kadariya Y , Cheung M , Xu J *et al*. Bap1 is a bona fide tumor suppressor: genetic evidence from mouse models carrying heterozygous germline Bap1 mutations. Cancer Res 2016; 76; 2836–2844. 26896281 10.1158/0008-5472.CAN-15-3371 PMC4873414

[16] Wang S , Gu YF , Wolff N *et al*. Bap1 is essential for kidney function and cooperates with Vhl in renal tumorigenesis. Proc Natl Acad Sci USA 2014; 111; 16538–16543. 25359211 10.1073/pnas.1414789111 PMC4246264

[17] Yu H , Pak H , Hammond‐Martel I *et al*. Tumor suppressor and deubiquitinase BAP1 promotes DNA double‐strand break repair. Proc Natl Acad Sci 2014; 111; 285–290. 24347639 10.1073/pnas.1309085110 PMC3890818

[18] Piris A , Mihm Jr MC , Hoang MP . BAP1 and BRAFV600E expression in benign and malignant melanocytic proliferations. Hum Pathol 2015; 46; 239–245. 25479927 10.1016/j.humpath.2014.10.015

[19] Aung PP , Nagarajan P , Tetzlaff MT *et al*. Melanoma with loss of BAP1 expression in patients with no family history of BAP1‐associated cancer susceptibility syndrome: a case series. Am J Dermatopathol 2019; 41; 167–179. 30801340 10.1097/DAD.0000000000001217 PMC8191382

[20] Marušić Z , Buljan M , Busam KJ . Histomorphologic spectrum of BAP1 negative melanocytic neoplasms in a family with BAP1‐associated cancer susceptibility syndrome. J Cutan Pathol 2015; 42; 406–412. 25902915 10.1111/cup.12493

[21] Xu Y , Gru AA , Brenn T , Wiedemeyer K . BRCA1‐associated‐protein‐1 inactivated melanocytic tumours: characterisation of the clinicopathological spectrum and immunohistochemical expression pattern of preferentially expressed antigen in melanoma. Histopathology 2024; 86; 294–301. 39268598 10.1111/his.15318 PMC11649512

[22] Wiesner T , Kutzner H , Cerroni L , Mihm MC Jr , Busam KJ , Murali R . Genomic aberrations in spitzoid melanocytic tumours and their implications for diagnosis, prognosis and therapy. Pathology 2016; 48; 113–131. 27020384 10.1016/j.pathol.2015.12.007 PMC4817351

[23] Massi D , Cesinaro AM , Tomasini C *et al*. Atypical Spitzoid melanocytic tumors: a morphological, mutational, and FISH analysis. J Am Acad Dermatol 2011; 64; 919–935. 21496703 10.1016/j.jaad.2010.05.043

[24] Gassenmaier M , Soltanpour N , Held L *et al*. Diagnostic and prognostic classification of atypical spitzoid tumours based on histology and genomic aberrations: A prospective cohort study with long‐term follow‐up. Eur J Cancer 2022; 163; 200–210. 35104769 10.1016/j.ejca.2021.12.016

[25] Hanauer DA , Mei Q , Law J , Khanna R , Zheng K . Supporting information retrieval from electronic health records: a report of University of Michigan's nine‐year experience in developing and using the Electronic Medical Record Search Engine (EMERSE). J Biomed Inform 2015; 55; 290–300. 25979153 10.1016/j.jbi.2015.05.003 PMC4527540

[26] Bastian BC , Olshen AB , LeBoit PE , Pinkel D . Classifying melanocytic tumors based on DNA copy number changes. Am J Pathol 2003; 163; 1765–1770. 14578177 10.1016/S0002-9440(10)63536-5 PMC1892437

[27] Chandler WM , Rowe LR , Florell SR , Jahromi MS , Schiffman JD , South ST . Differentiation of malignant melanoma from benign nevus using a novel genomic microarray with low specimen requirements. Arch Pathol Lab Med 2012; 136; 947–955. 22849744 10.5858/arpa.2011-0330-OA

[28] Wang L , Rao M , Fang Y *et al*. A genome‐wide high‐resolution array‐CGH analysis of cutaneous melanoma and comparison of array‐CGH to FISH in diagnostic evaluation. J Mol Diagn 2013; 15; 581–591. 23800576 10.1016/j.jmoldx.2013.04.001

[29] Ardakani NM , Thomas C , Robinson C *et al*. Detection of copy number variations in melanocytic lesions utilising array based comparative genomic hybridisation. Pathology 2017; 49; 285–291. 28274670 10.1016/j.pathol.2016.11.008

[30] Yazdan P , Cooper C , Sholl LM *et al*. Comparative analysis of atypical spitz tumors with heterozygous versus homozygous 9p21 deletions for clinical outcomes, histomorphology, BRAF mutation, and p16 expression. Am J Surg Pathol 2014; 38; 638–645. 24451276 10.1097/PAS.0000000000000160

[31] Gerami P , Busam K , Cochran A *et al*. Histomorphologic assessment and interobserver diagnostic reproducibility of atypical spitzoid melanocytic neoplasms with long‐term follow‐up. Am J Surg Pathol 2014; 38; 934–940. 24618612 10.1097/PAS.0000000000000198

[32] Ludgate MW , Fullen DR , Lee J *et al*. The atypical Spitz tumor of uncertain biologic potential: a series of 67 patients from a single institution. Cancer 2009; 115; 631–641. 19123453 10.1002/cncr.24047

[33] Lallas A , Kyrgidis A , Ferrara G *et al*. Atypical Spitz tumours and sentinel lymph node biopsy: a systematic review. Lancet Oncol 2014; 15; e178–e183. 24694641 10.1016/S1470-2045(13)70608-9

[34] Linos K , Atkinson AE , Yan S , Tsongalis GJ , Lefferts JA . A case of molecularly confirmed BAP1 inactivated melanocytic tumor with retention of immunohistochemical expression: a confounding factor. J Cutan Pathol 2020; 45; 485–489. 10.1111/cup.13642 PMC10860368 31891422

